# Serum Markers of Hepatocyte Death and Apoptosis Are Non Invasive Biomarkers of Severe Fibrosis in Patients with Alcoholic Liver Disease

**DOI:** 10.1371/journal.pone.0017599

**Published:** 2011-03-18

**Authors:** Vanessa Jeannette Lavallard, Stéphanie Bonnafous, Stéphanie Patouraux, Marie-Christine Saint-Paul, Déborah Rousseau, Rodolphe Anty, Yannick Le Marchand-Brustel, Albert Tran, Philippe Gual

**Affiliations:** 1 Team 8, ≪Hepatic complications of obesity≫, INSERM, U895, Nice, France; 2 Faculty of Medicine, University of Nice-Sophia-Antipolis, Nice, France; 3 Digestive Center, Centre Hospitalier Universitaire of Nice, Nice, France; 4 Biological Center, Centre Hospitalier Universitaire of Nice, Nice, France; Chinese University of Hong Kong, Hong Kong

## Abstract

**Background:**

Quantification of hepatotocyte death is useful to evaluate the progression of alcoholic liver diseases. Our aims were to quantify and correlate the circulating levels of Cytokeratin 18 (CK18) and its caspases-generated fragment to disease severity in heavy alcoholics.

**Methodology/Principal Findings:**

CK18 and CK18-fragment were evaluated in the serum of 143 heavy alcoholics. Serum levels of markers of hepatocyte death (CK18), apoptosis (CK18 fragment) and necrosis (CK18 -CK18 fragment) increased in patients with severe fibrosis compared to patients with mild fibrosis. These markers strongly correlated with Mallory-Denk bodies, hepatocyte ballooning, fibrosis and with hepatic TNFα and TGFβ assessed in the liver of 24 patients. Elevated levels of serum hepatocyte death and apoptotic markers were independent risk factors in predicting severe fibrosis in a model combining alkaline phosphatase, bilirubin, prothrombin index, hyaluronate, hepatocyte death and apoptotic markers. The level of markers of hepatocyte death and apoptosis had an area under the receiving operator curve that predicted severe fibrosis of 0.84 and 0.76, respectively.

**Conclusion/Significance:**

Death of hepatocytes can be easily evaluated with serum markers and correlated with severe fibrosis in heavy alcohol drinkers. These biomarkers could be useful to rapidly evaluate liver injuries and the efficacy of therapies.

## Introduction

Excessive alcohol consumption is the third leading preventable cause of death in the United States [1]. Regular alcohol use can result in hepatic steatosis, which eventually progresses to steatohepatitis, fibrosis and cirrhosis. Up to 40% of patients with severe acute alcoholic hepatitis die within six months [1]. A large body of evidence suggests that apoptosis of hepatocytes may be a key mechanism of alcohol-induced liver injury [2], [3]. It has been largely reported that apoptosis of hepatocytes is a significant histological feature of human Alcoholic Liver Diseases (ALD). The magnitude of apoptosis evaluated by the Tunel assay, caspase activation and the apoptotic index correlated with the severity of ALD, the degree of inflammation and stage of fibrosis [2]. Hepatocyte apoptosis is more pronounced in patients with high bilirubin and AST levels [2]. Death receptors (i.e. TNFα/TNFα Receptor, Fas-L/Fas), oxidative and endoplasmic reticulum stress, glutathione depletion could play an important role in alcohol induced apoptosis of hepatocytes [2], [3]. Elevated circulating levels of soluble Fas, Fas ligand and TNFα have been reported [4], [5], together with upregulation of the receptors in patients with ALD [6], [7], [8]. When the activation of mitochondrial-dependent apoptosis was more severe and involved most of the mitochondria, ATP was depleted and could result in a switch from apoptosis to necrosis [9]. Cytokeratin 18 (CK18) is the major intermediate filament protein in the liver and one of the most prominent substrates of caspases during apoptosis of hepatocytes [10]. Caspases-generated CK18 fragments are released from the tissue and are resistant to proteolysis [11], [12]. CK18 is cleaved by caspases at two sites (Asp238 and Asp396) and the M30 antibody recognizes the neo-epitope mapped to positions 387 to 396, which is only revealed after caspase cleavage. The M30-based ELISA thus determines the circulating levels of a specific caspases-generated CK18 fragment and is proposed as a surrogate biomarker of cell apoptosis [11]. The M65-based ELISA determines the circulating levels of both the caspases-generated fragment and intact CK18. The soluble full-length CK18 is released from cells undergoing necrosis [11], [12]. These markers have been evaluated in patients with different chronic liver diseases including alcoholic and nonalcoholic fatty liver diseases [13]. The CK18 fragment level appears to be a noninvasive biomarker for nonalcohololic steatohepatitis [14], [15], [16], [17] and changes in the level correlate with changes in the NAFLD activity score [18]. In contrast, few studies have been performed on patients with ALD. The level of CK18 or of the CK18 fragment was frequently increased in small groups of alcoholics and may act as a marker of hepatitis [19], [20], [21]. The serum levels of total CK18 in heavy drinkers (n = 15) were higher than those of healthy controls (n = 10), and even tended to be higher than those of patients with biopsy-proven malignancy of epithelial origin (n = 22) including adenocarcinoma of a variety of origins, small lung carcinoma, hypernephroma and epidermoid easophageal carcinoma [22]. The same group showed that serum levels of total CK18 correlated with the apoptotic score in 53 patients (31 with alcoholic hepatitis and 22 with fatty liver) [19]. More recently, it has been reported that serum levels of CK-18 fragment in patients with alcoholic hepatitis (n = 50) were higher than those of healthy controls (n = 50) and heavy drinkers (n = 50), and even tended to be higher than those of patients with malignancy (n = 50) [21].

In this study, we focused on the death of hepatocytes by apoptosis and/or necrosis as evaluated by circulating serum biomarkers in a cohort of 143 patients with ALD and investigated correlation with disease severity. The prediction of severe fibrosis based on these markers was also evaluated.

## Materials and Methods

### Study population

From October 1997 to June 1998, 143 consecutive heavy alcohol drinkers (105 men, 38 women, mean age: 48±9 years) admitted to our Liver unit for detoxification and/or in patient rehabilitation were entered into this study. All patients had consumed over 80 g ethanol per day for more than 5 years. All patients were negative for circulating hepatitis B surface antigen, hepatitis C virus, and human immunodeficiency virus. No patient had osteoarthritis or rheumatoid arthritis. Fasting blood samples were used to measure alanine amino transferase (ALT), aspartate aminotransferase (AST) and gamma glutamyl transferase (γGT). A needle liver biopsy was performed on all patients by the transparietal approach. Biopsies were processed routinely and stained with hematoxylin-eosin-safran and Sirius Red. The length of the liver biopsy was over 15 mm. Histopathological features were semi-quantitatively evaluated: grade of steatosis (0, <5%; 1, 5%–30%; 2, >30%–60%; 3, >60%); hepatocellular ballooning (0, none; 1, few balloon cells; 2, many cells/prominent ballooning); megamitochondria (0, none to rare; 1, many); Mallory's hyaline (0, none to rare; 1, many) and fibrosis stage (From 0, none, to 4, cirrhosis). The grading of hepatic activity was done according to the Activity score of Orrego et al. [23]. The characteristics of whole cohort from which a representative group of 24 alcoholic patients was selected for the hepatic gene expression analysis are described in Table 1. Sera and liver tissues were stored at −80°C until use. These patients were included later in the “Fibroscore program”. All subjects gave their informed written consent to participate in this research study according to French legislation regarding Ethic and Human Research (Huriet-Serusclat law, the “Comité Consultatif de Protection des Personnes dans la Recherche Biomédicale de Nice” approved this study, N°03.613).

**Table 1:** Characteristics of Serum and Gene groups.

	Serum group	Gene group
**N (female/male)**	143 (38/105)	24(4/20)
**Age (years)**	48.0(42.0, 53.0)	48.0(46.0, 53.0)
**Alcohol (g/day)**	120.0(90.0, 182.5)	142.0(90.0, 170.0)
**AST (IU/L)**	66.0(41.0, 120.0)	52.0(32.0, 119.0)
**ALT (IU/L)**	41.0(25.0, 78.0)	39.0(23.0, 57.5)
**AST/ALT**	1.7(1.1, 2.5)	1.7(1.2, 2.6)
**γGT (IU/L)**	181.0(98.0, 394.0)	137.0(83.0, 514.0)
**Steatosis (%)**		
**<5%**	7.6	4.2
**5–30%**	59.0	66.7
**>30–60%**	25.7	12.5
**>60%**	11.4	16.7
**Inflammation (%)**	22.9	16.7
**Mallory-Denk body (%)**	35.2	16.7
**Ballooning (%)**	32.4	45.8
**Fibrosis (%)**		
**0**	11.2	8.3
**1**	37.8	41.7
**2**	10.5	16.7
**3**	36.4	8.3
**4**	4.2	25.0

Data are expressed as Median (25th, 75th percentile) or %. AST: aspartate amino-tranferase; ALT: alanine amino-transferase; γGT: Gamma Glutamyl Transpeptidase.

### Circulating levels of total and fragmented CK18

Intact CK18 is released by necrotic cells. CK18 is also cleaved by caspases during apoptosis, generating soluble protein fragments. The M65® ELISA assay, which detects all forms of CK18, measures cell death due to both apoptosis and necrosis (CK18 intact and fragment), while the M30 Apoptosense® ELISA assay specifically measures apoptosis (the caspases-generated CK18 fragment, CK18-Asp396). The quantification of necrosis results from the difference between total CK18 values (M65) and the values of the caspases-generated fragment (M30). The caspases-generated CK18 fragment (M30 antigen) and CK18 (M65 antigen) were evaluated in the serum of 143 patients with M30 Apoptosense® ELISA and M65® ELISA kits (PEVIVA), respectively, as described by the manufacturer's instructions. All samples were analyzed in duplicate, in random order and blinded to the clinical/pathological data. For both the M30 Apoptosense® ELISA and M65® ELISA, within assay (WA % CV) variation was <10% and between assay (BA % CV) variation was <10% for samples >100 U/L. The minimum detectable concentration of M30 and M65 were 25 U/L and 11 U/L, respectively. Cytokeratins are released into the circulation as protein complexes. These complexes are remarkably stable during sample collection and long-term storage. Furthermore, plasma/serum samples can be exposed to repetitive freeze-thaw cycles without loss of activity [24].

### Real-time quantitative PCR analysis

Total RNA was extracted from human tissues using a RNeasy Mini Kit (Qiagen, Contraboeuf, France). The samples were treated with Turbo DNA-free™ (Applied Biosystems, Contraboeuf, France) following the manufacturer's protocols. The quantity and quality of the isolated RNA were determined using the Agilent 2100 Bioanalyser with RNA 6000 Nano Kit (Agilent Technologies). Total RNA (0.5 µg) was reverse-transcribed with the High Capacity cDNA Reverse Transcription Kit (Applied Biosystems, Foster City, CA). Real-time quantitative PCR was performed using the ABI PRISM 7500 Fast Real Time PCR System and FAM™ dyes (Applied Biosystems, Contraboeuf, France) following the manufacturer's protocols in our Genomics facilities. The TaqMan® gene expression assays were purchased from Applied Biosystems: Fas-L, Hs00181225_m1; TNFα, Hs00174128_m1; TGFβ, Hs00171257_m1; and RPLP0; Hs99999902_m1. Gene expression values were normalized to the value of the housekeeping gene *RPLP0* (Ribosomal Phosphoprotein Large P0) and calculated based on the comparative cycle threshold Ct method (2^−ΔΔCt^), as previously described [25], [26]

### Statistical analysis

Statistical significance of the differential circulating levels of the liver markers of total hepatocyte death, apoptotis and necrosis in patients with hepatic inflammation (A1) and severe fibrosis (F3/F4) compared to patients with no inflammation (A0) and to moderate fibrosis (F0/1/2), respectively, was determined using the non-parametric Mann-Whitney test. P<0.05 was considered as significant. Correlations were analyzed using the Spearman's rank correlation test. Comparisons were done using the Chi2 test or Fischer's exact tests for nominal data and by two-sample t tests for continuous data. Multivariate analyses were performed using binary logistic regression with estimation of odds ratios (OR) and 95% confidence intervals (95%CI). Diagnostic performance was determined by constructing a “receiver-operating characteristic” (ROC) curve and calculating the area under the ROC (AUROC) curve for prediction of patients with advanced fibrosis (F≥3) for serum cell death markers. From these curves, the best cut-off values were established for the serum cell death markers, which were the values that maximized the sum of the sensitivity and specificity to identify patient status. Statistical analyses were made using NCSS 2007 software.

## Results

### Elevated serum levels of total cell death, apoptotic and necrotic biomarkers correlated with hepatic inflammation, Mallory-Denk bodies, hepatocyte ballooning and fibrosis

**Figure 1:**
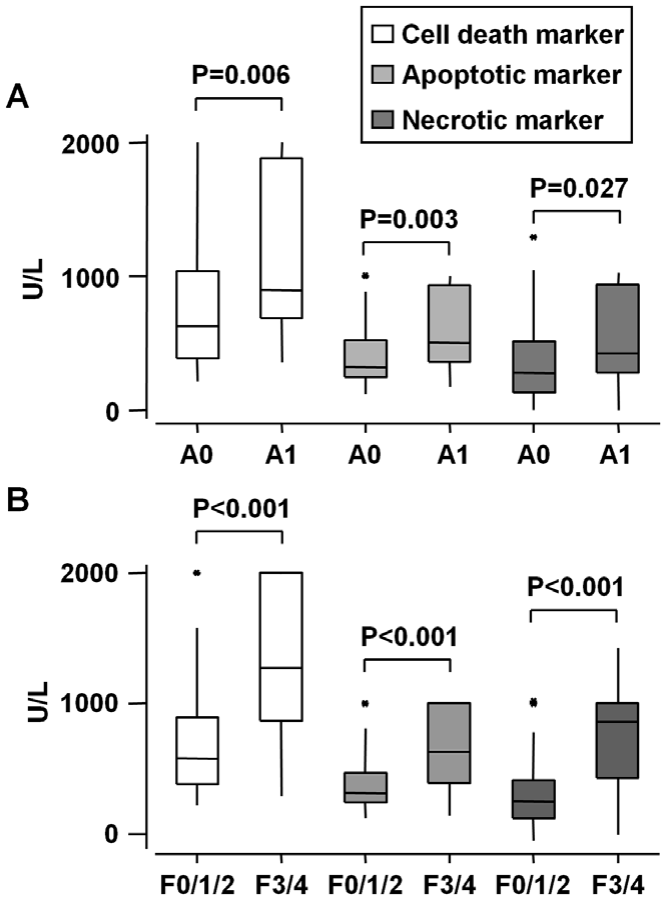
Elevated serum levels of total, apoptotic and necrotic cell death markers in patients with hepatic inflammation and advanced fibrosis. The serum of 143 alcoholic patients were used to evaluate the circulating levels of total CK18 (M65® ELISA) and the caspases-generated CK18 fragment (M30 Apoptosense® ELISA). Results were expressed as median (25^th^, 75^th^ percentile) in function to: (A) hepatic inflammation (A1) and (B) advanced fibrosis (F3/4).

The goal of this study was to better characterize the hepatic apoptosis and necrosis induced by chronic alcohol consumption. To quantify liver cell death by apoptosis and/or necrosis in alcoholic patients, we evaluated the circulating levels of biomarkers of cell death (soluble intact CK18 or the caspases-generated CK18 fragment) in 143 alcoholic patients without or with hepatic inflammation and with different grades of fibrosis (Table 1). As shown in Figure 1, the circulating levels of the liver markers of total hepatocyte death, apoptotis and necrosis were increased in patients with hepatic inflammation (A1) and severe fibrosis (F3/F4) compared to patients with no inflammation (A0) and to moderate fibrosis (F0/1/2), respectively. Further, the three biomarkers positively correlated with hepatic inflammation, Mallory-Denk bodies, ballooning and fibrosis (Table 2).

**Table 2:** Correlation between circulating levels of total, apoptotic and necrotic cell death markers and hepatic inflammation, Mallory-Denk bodies, ballooning and fibrosis in 143 alcoholic patients.

Marker of	Inflammation		Mallory-Denk bodies		Ballooning		Fibrosis	
	r_s_	P	r_s_	P	r_s_	P	r_s_	P
**Cell death (CK18 total)**	0.284	0.003	0.493	<0.001	0.352	<0.001	0.617	<0.001
**Apoptosis (CK18 fragment)**	0.297	0.002	0.496	<0.001	0.330	0.001	0.472	<0.001
**Necrosis (CK18 total-fragment)**	0.238	0.012	0.426	<0.001	0.316	0.001	0.594	<0.001

Spearman's rank correlation test.

### Elevated total cell death, apoptotic and necrotic markers correlate with hepatic expression of TNFα and TGFβ

In patients with ALD, liver apoptotic cell death and fibrosis were reported to be associated with upregulation of TNFα, Fas-L and TGFβ, respectively [4], [5], [6], [7], [8], [27]. So we evaluated the expression of their mRNA level in the liver of 24 alcoholic patients. As expected, the gene expression of TNFα, Fas-L and TGFβ was increased in patients with severe fibrosis (F3/F4) compared with patients with no or mild fibrosis (F0/F1)(TNFα, 1.74±0.27 vs 1±0.16, P = 0.039; Fas-L, 1.55±0.13 vs 1±0.11, P = 0.007; TGFβ, 2.33±0.17 vs 1±0.16, P = 0.0001) and correlated with the grade of fibrosis (Table 3). Furthermore, the serum markers of total, apoptotic and necrotic cell death correlated with hepatic expression of TNFα and TGFβ (Table 3).

**Table 3:** Correlation between hepatic TGFβ, TNFα and Fas-L gene expression and circulating levels of total, apoptotic and necrotic cell death markers in 24 alcoholic patients.

	Fibrosis		Cell death marker(CK18 total)		Apoptotic marker(CK18 fragment)		Necrotic marker(CK18 total-fragment)	
	r_s_	P	r_s_	P	r_s_	P	r_s_	P
TGFβ	0.792	<0.001	0.589	0.005	0.547	0.010	0.631	0.002
TNFα	0.489	0.034	0.519	0.019	0.504	0.033	0.529	0.016
Fas-L	0.577	0.008	0.359	0.120	0.347	0.134	0.283	0.226

The correlation between the expression levels of TGFβ, TNFα and Fas-L mRNA (ΔCt) and circulating levels of biomarkers was analyzed using the Spearman's rank correlation test.

### The serum total cell death and apoptotic markers were independent risk factors in predicting severe liver fibrosis in patients with alcoholic liver diseases

Since the circulating level of total cell death and apoptotic markers was higher in patients with severe fibrosis (F3) compared with patients with moderate fibrosis (F2) (data not shown), we investigated the pertinence of the levels of serum total cell death and apoptotic markers in predicting severe fibrosis. Using the univariate analysis (Table 4), patients with advanced fibrosis (F≥3) were older with higher levels of total cell death, apoptotic and necrotic markers, alkaline phosphatase, bilirubin and hyaluronate levels and lower levels of platelets, albumin and of the prothrombin index compared to patients with no to moderate fibrosis (F<3) (Table 4). In contrast, the gender, the AST, ALT and γGT levels were not associated with F≥3. In a multivariate analysis including total cell death and apoptotic markers, alkaline phosphatase, bilirubin, prothrombin index and hyaluronate levels, the only independent variables, when the judgment criterion was F ≥3, were prothrombin index, hyaluronate, total and apoptotic cell death markers (Table 5).

**Table 4:** Univariate analysis of the 143 alcoholic patients according to the severity of liver disease.

Data	F<3 (n = 85)	F≥3 (n = 58)	P
**Age (years)**	45.8±7.8	51.2±8.8	<0.000001
**Gender (female/male)**	19/66	19/39	0.166613
**Platelets (10^9^/L)**	204.0±77.6	132.6±76.2	<0.000001
**AST (IU/L)**	90.4±103.5	106.6±81.6	0.453246
**ALT (IU/L)**	79.6±175.3	41.4±31.8	0.102965
**γGT (IU/L)**	302.2±359.1	368.6±391.1	0.296977
**Alkaline phosphatase (IU/L)**	88.8±39.4	163.6±89.8	<0.000001
**Bilirubin (µmol/L)**	10.3±6.2	77.6±93.9	<0.000001
**Albumin (g/L)**	47.5±4.5	31.6±8.6	<0.000001
**Prothrombin index (%)**	97.3±5.6	60.1±20.3	<0.000001
**Total cell death marker (U/L)**	669.6±397.2	1392.2±566.4	<0.000001
**Apoptotic marker (U/L)**	381.6±208.3	658.4±305.2	<0.000001
**Necrotic marker (U/L)**	294.7±237.6	733.8±335.6	<0.000001
**Hyaluronate (µg/L)**	43.5±31.3	486.1±305.6	<0.000001

Patients were classified as Fibrosis (F) <3 or ≥3. Quantitative results are expressed as means ± standard deviations. AST: aspartate amino-tranferase; ALT: alanine amino-transferase; γGT: Gamma Glutamyl Transpeptidase.

**Table 5:** Multivariate analysis for the prediction of the severity of liver disease.

Data	F≥3 versus F<3		
	P	OR	95%CI
**Alkaline phosphatase**	0.1566	0.901	0.9556–1.0072
**Bilirubin**	0.3540	0.902	0.7273–1.1207
**Prothrombin index**	0.0142	0.675	0.4940–0.9245
**Cell death marker**	0.0497	0.991	0.9833–0.9999
**Apoptotic marker**	0.0211	1.032	1.0047–1.0607
**Hyaluronate**	0.0073	1.068	1.0181–1.1221

Patients were classified according to Fibrosis (F) <3 or ≥3. Multivariate analysis was performed using logistic regression.

### The three biomarkers of cell death accurately predicted severe fibrosis in alcoholic patients

**Figure 2:**
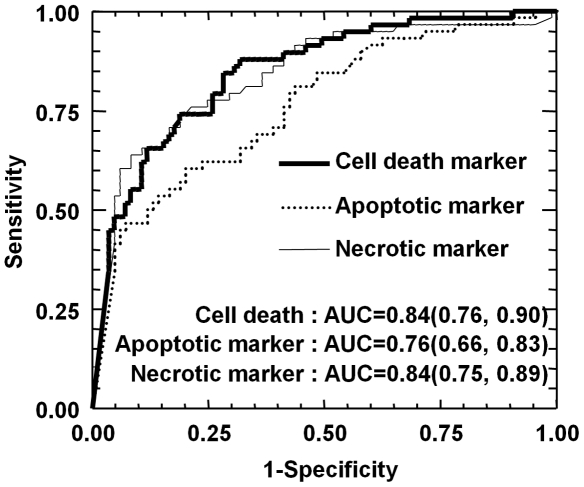
Levels of total, apoptotic and necrotic cells death markers for diagnosis of advanced fibrosis (F≥3) in 143 alcoholic patients. The area under the ROC curves are shown for the performance of the total (CK18 total), apoptotic (CK18 fragment) and necrotic (CK18 total-fragment) cell death markers for predicting advanced fibrosis (F≥3).

We then wanted to determine if these serum biomarkers could predict severe fibrosis (F≥3) in our cohort of 143 patients. As shown in Figure 2, the area under the ROC curve (AUC) for discriminating severe fibrosis with the total cell death, apoptotic or necrotic markers was 0.84 (CI95% 0.76, 0.90), 0.76 (CI95% 0.66, 0.83) and 0.84 (CI95% 0.75, 0.89), respectively. The comparison of the AUC indicated that the total cell death marker is significantly different from the apoptotic marker (total cell death marker AUROC versus apoptotic marker AUROC: *P* = 0.0006; total cell death marker AUROC versus necrotic marker AUROC: *P* = 0.73; apoptotic marker AUROC versus necrotic marker AUROC: *P* = 0.0505).

Several cut-off values were calculated for the levels of the three markers for the prediction of F≥3 in our cohort (Table 6). For example, the cut-off point at 790 U/L of the cell death marker predicted F≥3 with a sensitivity of 84%, a specificity of 71%, a Positive and Negative Predictive Value of 67.1% and 87.1%, respectively. The cell death markers predicted severe fibrosis with good specificity and sensitivity indicating that hepatic cell death could be an important actor or characteristic of liver fibrosis induced by chronic alcohol consumption.

**Table 6:** Total, apoptotic and necrotic cell death marker levels for prediction of severe fibrosis (F≥3) (n = 143).

Cut-off ValueMarker of	Sensitivity	Specificity	LikelihoodRatio	PPV(Prev. 0.41)	NPV(Prev. 0.41)
**Cell death** **(CK18 total)**					
790	0.844	0.717	2.992	0.671	0.871
844	0.793	0.741	3.064	0.676	0.840
952	0.741	0.811	3.938	0.728	0.821
**Apoptosis** **(CK18 fragment)**					
346	0.810	0.564	1.861	0.559	0.813
415	0.706	0.611	1.820	0.554	0.753
500	0.603	0.800	3.017	0.673	0.747
**Necrosis** **(CK18 total-fragment)**					
380	0.810	0.670	2.459	0.626	0.838
430	0.758	0.788	3.582	0.709	0.827
511	0.706	0.835	4.291	0.745	0.806

PPV: Positive Predictive value, NPV: Negative Predictive value; Prev: Prevalence.

## Discussion

We report here that severe fibrosis associated with chronic alcoholic liver disease correlated with substantial liver cell death due to both apoptosis and necrosis, as evaluated by circulating levels of cell death markers. These serum markers also correlated with hepatic features of hepatocyte injury including ballooning and the presence of Mallory-Denk bodies and hepatic expression of TNFα and TGFβ. Finally, we show that the total, apoptotic and necrotic cell death markers accurately predict severe fibrosis in a large cohort of alcoholic patients.

Markers of total, apoptotic and necrotic cell death correlated with fibrosis and hepatic expression of TNFα and TGFβ in our patients. In agreement with our results, it has been shown that apoptosis of hepatocytes was significantly increased in patients with alcoholic hepatitis, and correlated with disease severity and hepatic fibrosis [27]. It has been proposed that alcohol alters the population of gut bacteria and inhibits intestinal motility, resulting in overgrowth of a Gram-negative flora. Lipopolysaccharide is elevated in portal blood and activates Kupffer cells leading to the production of ROS and, consequently, TNFα. TNFα then stimulates mitochondrial oxidant production in hepatocytes, which are sensitized to apoptosis [28]. Hepatic apoptosis produces chemokines and inflammation, which in turn may activate hepatic stellate cells. Moreover, the phagocytosis of hepatocyte apoptotic bodies by hepatic stellate cells and Kupffer cells enhances the expression of pro-fibrogenic genes, such as TGFβ, that may initiate hepatic fibrosis [27]. Although the degradation of cytokeratin during apoptosis is not completely understood, it has been suggested that it could facilitate the formation of apoptotic bodies and amplify the apoptotic signal [29]. We also showed that necrosis of hepatocytes was enhanced in severe fibrosis and could amplify these processes by massive release of cytokines.

Previous studies have shown that the serum levels of total CK18 (evaluated by a M3-based ELISA, the M3 antibody recognises soluble fragments of CK18) in heavy drinkers (n = 15) were higher than those of healthy controls (n = 10), and even tended to be higher than those of patients with biopsy-proven malignancy of epithelial origin (n = 22) including adenocarcinoma of a variety of origins, small lung carcinoma, hypernephroma and epidermoid easophageal carcinoma [22]. The same group showed that serum levels of total CK18 correlated with the apoptotic score in 53 patients (31 with alcoholic hepatitis and 22 with fatty liver) [19]. While these studies were done on a small group of patients, the circulating level of total CK18 was increased in alcoholics. We now reported that circulating levels of these cell death markers correlated better with fibrosis than with the hepatic activity in a large cohort of alcoholic patients (n = 143).

We have shown for the first time that the serum cell death and apoptotic markers were independent factors that predict severe fibrosis in a large cohort of patients with ALD. Liver biopsy remains the gold standard for assessment of liver fibrosis. However, problems with liver biopsy include sampling error and inter-observer variability. Fibroscan and the currently available sero-algorithm tests (Fibrotest®, Fibrometer®) or the direct biomarker (Hyaluronate) can differentiate between mild and severe disease [30], [31], [32]. In the case of borderline results two or more methods can be combined. However, identification of novel markers is needed to improve sero-algorithm tests leading to quantification of fibrosis and to monitor the dynamic nature of fibrosis.

Cell death biomarkers have already been inserted into a composite model for prediction of liver injury or patient survival in other liver diseases. In acute liver failure, the cytokeratin 18-based modification of the model for the End-Stage Liver Disease (MELD) score improves prediction of spontaneous survival after acute liver injury [33]. We have recently shown that the association of serum CK18 fragments with ALT and the presence of a metabolic syndrome in a composite model predicted hepatic inflammation in morbidly obese patients [34]. These markers could also be useful in rapidly providing information concerning the treatment response. For example, the apoptotic cell death (CK18 fragment) marker has been recently used to evaluate the effect of some treatments in patients with chronic hepatitis C infection [35], [36].

In summary, circulating levels of total and the caspases-generated fragment of cytokeratin 18 predict with good accuracy severe fibrosis in heavy alcohol drinkers. These markers also correlated with hepatocyte ballooning, the presence of Mallory-Denk bodies and hepatic TNFα and TGFβ expression. Furthermore, studies focusing on the behavior of these markers for the follow up of patients with severe alcoholic liver disease should be of great interest, particularly in response to corticosteroid.
